# Supplementary figures and images for: Exogenous Nitro-Oleic Acid Treatment Inhibits Primary Root Growth by Reducing the Mitosis in the Meristem in Arabidopsis thaliana
Source: Front Plant Sci. 2020 Jul 21;11:1059. doi: 10.3389/fpls.2020.01059 (PMC7385231; doi:10.3389/fpls.2020.01059)

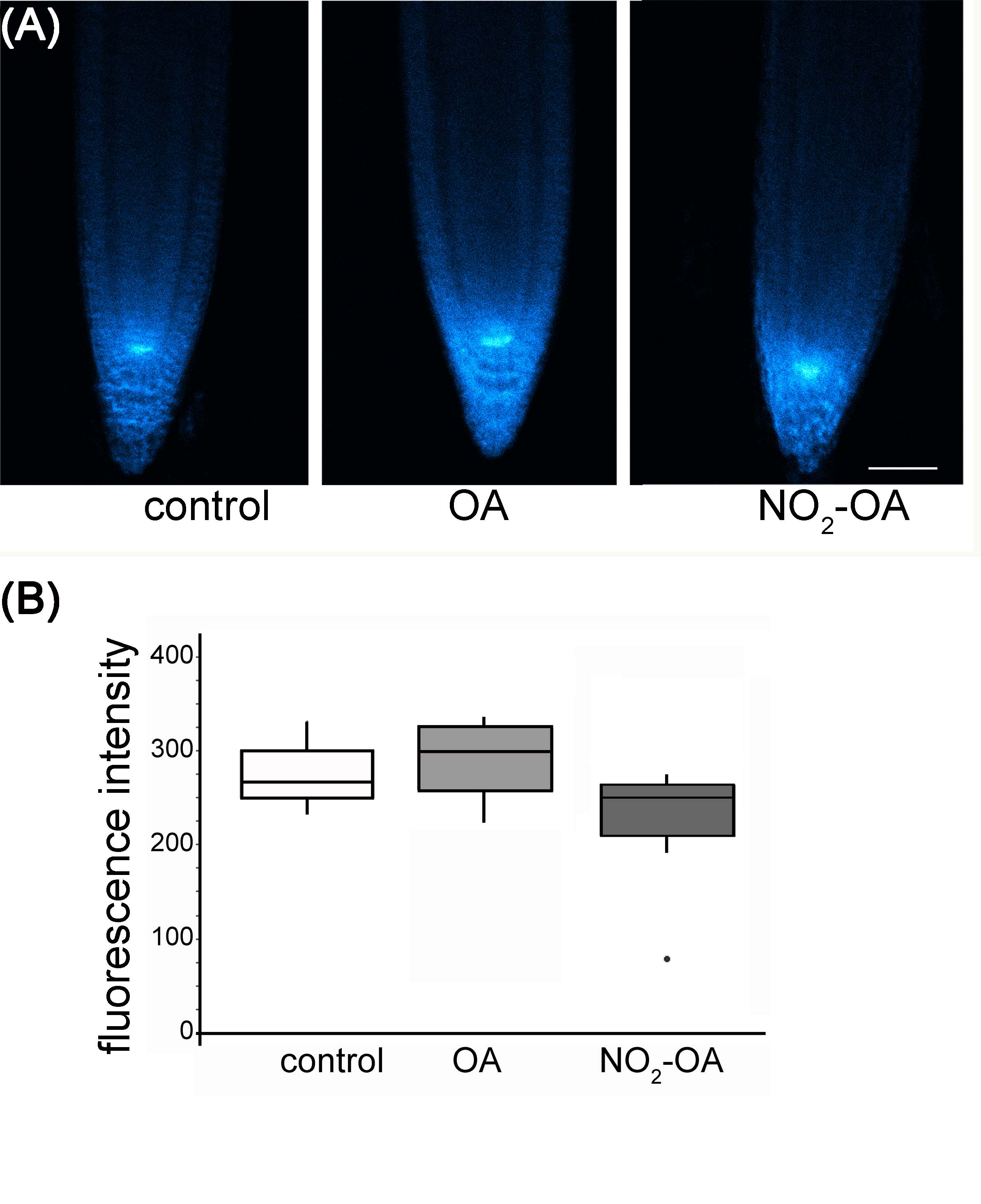

Supplement: Supplemental Figure S1 — Effect of NO2-OA on the expression of PLT2 transcription factor in Arabidopsis roots. Seedling of proPLT2::CFP reporter line were grown for 5 days and then treated with NO2-OA or OA (12.5 μM) or not treated during other five days. Representative confocal images are shown. Fluorescence intensity measured from at least 12 roots from 3 independent experiments. Scale bars = 10 μm. [file Image_1.jpeg]
