# Supplementary material for: Exogenous Nitro-Oleic Acid Treatment Inhibits Primary Root Growth by Reducing the Mitosis in the Meristem in Arabidopsis thaliana
Source: Front Plant Sci. 2020 Jul 21;11:1059. doi: 10.3389/fpls.2020.01059 (PMC7385231; doi:10.3389/fpls.2020.01059)
Supplement: Supplementary file 2 [file Table_1.doc]

**Legend**

Supplemental Figure S1:

Effect of NO_2_-OA on the expression of PLT2 transcription factor in Arabidopsis roots. Seedling of *proPLT2::CFP* reporter line were grown for 5 days and then treated with NO_2_-OA or OA (12.5 μM) or not treated during other five days. Representative confocal images are shown. Fluorescence intensity measured from at least 12 roots from 3 independent experiments. Scale bars = 10 μm.
